# Exploring Health-Related Quality of Life (HRQOL) among patients with HIV-associated TB in Khayelitsha, South Africa

**DOI:** 10.1371/journal.pone.0275554

**Published:** 2024-11-04

**Authors:** John-Henry Hickman, Alison Swartz, Namhla Sicwebu, Cari Stek, Nobom Masimini, Christiana Nöstlinger

**Affiliations:** 1 Division of Social and Behavioural Sciences, School of Public Health and Family Medicine, University of Cape Town, Cape Town, South Africa; 2 St George’s School of Health and Medical Sciences, City St George’s University of London, London, United Kingdom; 3 Department of Clinical Sciences, Institute of Tropical Medicine, Antwerp, Belgium; 4 Wellcome Centre for Infectious Diseases Research in Africa, Institute of Infectious Disease and Molecular Medicine, University of Cape Town, Cape Town, South Africa; 5 Department of Public Health, Institute of Tropical Medicine, Antwerp, Belgium; University of Greenwich, UNITED KINGDOM OF GREAT BRITAIN AND NORTHERN IRELAND

## Abstract

**Background:**

Health-related quality of life (HRQOL) is an important and frequently used patient-reported outcome in health research. However, little qualitative research exists in this field in South Africa. This study was set in Khayelitsha, one of the largest informal settlements in South Africa, where the burden of HIV and tuberculosis (TB) co-infection are amongst the highest in the world and significantly affect HRQOL.

**Objective:**

To explore the experience of HRQOL among patients living with HIV-associated TB.

**Methods:**

We conducted sixteen interviews with male (n = 10) and female (n = 6) adult participants (ages 24–56; median age 35) to explore their HRQOL living with HIV-associated TB, related treatment and how this impacted on life domains they considered relevant for HRQOL. We used thematic analysis to analyse data, using both an inductive and deductive analysis using an interpretive phenomenological approach (IPA).

**Results:**

Experiences of HRQOL were identified along the predominantly emerging themes of physical, social, and mental aspects of HRQOL. Identified sub-themes included well-being, loss of strength, and self-care for the physical domain; usual activities and stigma for the social domain; concerns and coping for the mental domain. The findings illustrate that HRQOL domains are interconnected through social experience. The social experience of HRQOL was identified as the common denominator connecting all domains, around which HRQOL revolved for these participants.

**Conclusion:**

HRQOL is experienced socially. The interpersonal connections patients have with significant others present as key to high HRQOL among patients living with HIV-associated TB. This study adds to the existing literature of HRQOL and examines HRQOL using IPA which may help to inform future interventions to improve HRQOL among HIV/TB patients.

## Introduction

Despite significant progress in addressing infections with Human Immunodeficiency Virus (HIV) and Tuberculosis (TB), their sustained high prevalence remains a public health concern, particularly in resource-constrained contexts in South Africa [1]. The prevalence of HIV and TB tend to be higher in urban townships, where people often live in very crowded conditions, and struggle to negotiate socio-economic precarity. The South African picture regarding HIV and TB is further complicated by high HIV co-infection rates that exceed 60% among those living with TB [1, 2] Due to this high co-infection rate, the simultaneous experience of HIV and TB infection warrants further exploration. Health- related quality of life (HRQOL) research often explores patient-reported outcome measures (PROMs) that can offer valuable insight and information on therapeutic interventions, health strategies and health policy development [3–5]. This patient-centred exploration of the experience of health is particularly important as the disjuncture between patients’ subjective experience of treatment and well-being and clinical improvements have been observed [3]. Exploring HRQOL addresses this gap and is thus an increasingly significant component of HIV-related research [3, 6, 7].

HRQOL is considered synonymous with individual experiences [8–10], as opposed to clinical outcomes associated with treatment and patient health [11–13]. Individual experiences of living with a chronic disease are unique; it thus follows that HRQOL as a construct is highly subjective. Quantitative approaches may be limited in their ability to fully grasp the meanings people attach to social phenomena related to health [14, 15], and thus the call has been made for qualitative exploration of HRQOL [16, 17]. Qualitative approaches allow for capturing a more nuanced and contextualised account of patient experiences, beyond understanding health-related issues solely at the individual level [4, 18].

The concept of HRQOL has been discussed as an explicit target for introducing PROMs to measure the well-being of people living with HIV beyond clinical outcomes. In literature predominantly from South Africa, but also sub-Saharan Africa, the so-called “4th 90” (i.e. 90% of all people with suppressed viral load levels should have a good HRQOL) [18] became quickly criticised as not considering the role that good HRQOL may play for all people living with HIV. Rather, HRQOL was proposed as a dimension cutting across all stages of the HIV continuum of care [19]. Despite the advances in HIV treatment, people living with HIV continue to experience higher multimorbidity and poorer HRQOL than those not living with HIV. HRQOL is influenced by high degrees of stigma and discrimination [18, 20]. Experiences of stigma and discrimination were also shown to be a barrier to HIV treatment [21], HIV viral suppression [22], self-reported mental well-being and HRQOL[23]. Ample evidence exists for challenges in HRQOL for people living with either HIV or TB [24, 25]. What is less understood is the experience of HRQOL for those grappling with HIV/TB co-infection and related treatment [26]—a gap in the literature we aim to address in this article.

In this article we used a qualitative approach to contribute to understandings of HRQOL in relation to HIV and TB coinfection among people living in a South African resource-limited context. Aiming to identifying the essential structure of participants’ lived experiences [27, 28], we explored what HRQOL meant to individuals within their specific life contexts, including their social networks. We also sought to understand how those who participated in this study perceived the impact of living with HIV-associated TB on several life domains they considered important for HRQOL.

### Conceptual framework

Many of the existing conceptualisations of HRQOL perceive health in isolation of other life domains such as economic position, religious or spiritual domains [13]. In this article, we used Ebrahim’s (1995) definition, that states that HRQOL is a combination of self-perceived aspects related to well-being in the presence of disease or treatment [8]. These self-perceived aspects include constructs of HRQOL which align with the World Health Organisation’s (WHO) definition of health. The constructs of HRQOL are discussed in terms of life domains. These domains vary throughout literature and broadly include, but are not limited to, the physical, mental and social spheres of life pertaining to the individual [3, 4, 7, 8, 29, 30]. Here we explored these life domains from an interpretive phenomenological approach (IPA) [31] to better understand how meaning is ascribed to the experiences that people have of managing TB and HIV and related treatment within their social realities [32, 33].

## Methods

### Study context: PredART trial

This qualitative study was embedded in a randomised, double-blind, placebo-controlled trial to assess the efficacy of prednisone to prevent tuberculosis-associated immune reconstitution inflammatory syndrome (TB-IRIS) [34]. HRQOL was one of the secondary endpoints of the trial. To contextualise the quantitative measurements used in the trial on the prophylactic use of prednisone (PredART trial), qualitative data were collected to acquire information on the subjective meaning of HRQOL among people living with HIV-associated TB [34]. The PredART trial enrolled 240 patients at high risk of TB-IRIS between August 2013 and February 2016, 120 in the prednisone arm and 120 in the placebo arm. Patients were followed up over a 12-week period with five planned follow-up visits to monitor the development of TB IRIS and drug safety. Follow-up visits were scheduled at week 1, week 2, week 4, week 8, and week 12 from antiretroviral therapy (ART) initiation [35].

#### Methodological approach

Since we were interested in how individual experiences of illness and disease may shape quality of life, we used IPA to gain an understanding of the phenomenon of HRQOL in the given context [31]. We aimed to identify patterns of how living with HIV and TB itself may shape HRQOL, rather than only collecting participants’ opinions and perspectives about HRQOL. In line with recent developments in phenomenological research approaches [28], we assume that the findings are a result of an exploratory process through interaction between participants and researchers (through interviewing and interpreting) to capture essential aspects of participants’ experiences embedded in a medically and socially challenging context.

### Study setting and population

Khayelitsha is a peri-urban informal settlement approximately 30km from the central business district of Cape Town, South Africa. During the trial period Khayelitsha was estimated to have a population size of approximately 400 000 people and represents a tenth of the entire Cape Town Metropolitan [36]. Among health districts in the Western Cape, Khayelitsha has the highest HIV prevalence [37]. An estimated 16% of the population is living with HIV; TB case notification is 917/100,000 per year and HIV coinfection among TB cases is 60% [38].

### Sampling/Participant selection

A total of 16 patients who were already enrolled into the PredArt study were purposefully selected based on age, sex and TB-IRIS diagnosis to have broad representation of participants in the qualitative sub-study. Patients were included based on their availability and willingness to engage in interviews concerning quality of life related to HIV-associated TB. Participants were recruited with the support of the clinic counsellor, who had extensive experience in counselling patients with HIV and TB.

### Data collection

Semi-structured interviews were conducted by a native isiXhosa speaking clinic counsellor (NM) trained in administering semi-structured interviews. The semi-structured interview guide contained questions addressing the following topics: Definitions and experiences of health, experiences of treatment, including side effects and the meaning of HRQOL. The topic guide that informed our semi-structured interviews is available in the S1 Appendix.

Interviews were held in a private room at trial site. Interviews were scheduled after the week 4 follow-up visit. They were conducted in the patients’ preferred language–which for all participants was isiXhosa. Interviews were audio recorded and later translated and transcribed from isiXhosa to English. The duration of interviews varied, with the majority lasting 25–35 minutes. The shortest interview was 17 minutes while the longest lasted just over one hour.

### Positionality and reflexivity

The main data collector (NM) in this study had significant experience working with HIV/TB patients in care and as participants in clinical trials in Khayelitsha in particular. AS is a trained social scientist and anthropologist and supervised the field work. NS recently submitted her PhD in Public Health, and is a qualitative researcher fluent in isiXhosa who could assist with data analysis. Data collectors were thus familiar with working with participants grappling with HIV/TB, as well as the economic and social pressures of living in a township, which informed their reflections on qualitative data and observations during the interviews. JHH obtained his master’s degree in Public Health as part of the data analytical team and main contributor to the qualitative study report. CN is a health psychologist and senior social scientist. She conceptualized and supervised the study. Because this study was nested in a clinical trial, time pressure and patients’ ill-health sometimes did not allow for interviewing participants at length. Nevertheless, researchers were able to engage meaningfully with the participants, resulting in rich and robust data. In interpretation of the data, we addressed the question whether knowing about the interviewers’ professional background as counsellors could have impacted on participants’ willingness to freely share their experiences, or how this may have might have shaped what they shared. It cannot be totally excluded that the setting of a clinical trials may have biased the findings in terms of social desirability. As a research team, we also discussed how the wider social environment may have influenced the data collection and the findings. Hence, the coding framework that served to guide the analysis and data interpretation stressed the social determinants of health underpinning participants’ concepts of HRQOL. The teams’ reflections informed an analytic strategy combining insights from academic team members (AS, JHH, CN, NS) with a detailed understanding of the issues at stake and methodological issues, combined with field- and clinical-based contextual and experiential insights (NM).

### Data analysis

We utilised both an inductive and deductive analytical approach from an interpretive phenomenological approach. This approach was chosen as it allowed us to explore and understand at “patterns in participants’ experiences, the ways in which they make meaning of those experiences, and interpreting those experiences within social and theoretical contexts”(p.3) [31]. This included undertaking the analysis at two levels: first *describing* phenomena as they were seen and explained by participants, and secondly, using these descriptions to consider how participants ascribe *meaning* to phenomena, or *interpret* them. This interpretative process was informed by participants’ perspective, but also by the authors’ epistemological orientations that shaped the analysis process.

We initiated the inductive approach with the process of familiarisation with data in line with a thematic analysis approach [39] analysing the data descriptively. We used a stepwise approach of familiarisation with the data and coding the data. The codebook was developed through an inductive iterative process by the first author and reviewed by two other authors (AS & NS). Thereafter, the codebook was used in the process of indexing transcripts, where text sections were linked to themes via codes. Similar indexed sections were pooled together forming thematic sections with verbatim text and summaries of experiences. Through this approach, we interpreted the data, generated themes and reviewed them as research team. At this point, we applied a deductive analytical approach, where we pooled thematic sections into three predominant domains—physical, social and mental. Under each of these domains, relevant sub-themes linked to participants’ experience were explored in more detail.

### Ethical considerations

Patients were recruited for individual interviews as part of a trial which had obtained ethics approval from the University of Cape Town Human Research Ethics Committee (HREC 136/2013), Institute of Tropical Medicine (ITM) Institutional Review Board (882/13), and the Antwerp University Hospital Ethical Committee (13/20/224). Separate written informed consent for this sub-study was obtained.

## Results

### Participant characteristics

We interviewed 16 participants, ten male and six female participants aged 24–56 years, with a median age of 34 years. More detailed demographic and clinical variable of participants in the trial have been described elsewhere [34]. Participant recruitment was restricted by the requirements and timelines of the qualitative study being nested within a treatment trial, therefore we could not aim for thematic data saturation. We faced challenges in data collection as participants were often very ill and thus not able to comfortably participate in an interview or did not have the time to remain at the clinic for the duration of the interview as they needed to return to their other daily activities. Participation in an interview was also in addition to the time it took for other forms of data collection for the clinical trial. Some participants were employed in occupations such as construction workers, store assistants, taxi drivers, security, and gardening. Others (n = 6) were not employed at the time of the study. See Table 1 below for participant characteristics.

**Table 1:** Participant characteristics.

Participant number	Age (years)	Gender (M/F)	Education	Employment status	Occupation
1	31	M	Unknown	Employed	Construction worker
2	28	F	Never schooled	Unemployed	Unknown
3	33	M	Unknown	Employed (sick leave)	Store assistant
4	56	M	Never schooled	Unemployed	Gardener
5	32	F	Unknown	Unemployed	Unknown
6	31	M	Unknown	Self-employed (not working)	Construction (Glass/window fitter)
7	48	F	Unknown	Unemployed	Unknown
8	31	M	Grade 10	Employed	Security guard
9	45	F	Unknown	Unemployed	Kitchen staff
10	24	F	Unknown	Self-employed	Landlord
11	34	M	Unknown	Employed (sick leave)	Unknown
12	43	M	Unknown	Unemployed	Taxi driver
13	35	F	Unknown	Employed	Admin
14	39	M	Unknown	Employed (fixed term contract)	Construction (stone crushing)
15	35	M	Unknown	Employed (sick leave)	Security guard
16	35	M	Primary school	Employed (sick leave)	Store assistant

### Physical experiences of HRQOL

Most participants equated the concept of “health” with physical, bodily wellness. This was often the first point of discussion among participants in relation to living with HIV and TB. Participants often spoke about physical health in binary terms, as either being healthy or not or falling ill or not:

“He/she is healthy, and hardly gets sick. She/he is alright and well” (Female, 48, P7); “when you are not sick” (Male, 31); and “Good health, like in the body” (Male, 31, P6).

It appeared essential to participants that good HRQOL required good physical health. One exception was a participant who expressed that to him a clear distinction exists between health and quality of life and that one can have good HRQOL despite living with a disease.

“It’s important to note that these are two different things. To live a good life is different from good health.” (Male, 31, P8)

#### Loss of physical strength

A decrease in HRQOL was mostly experienced as a loss of physical strength. The loss of physical strength meant many participants could not engage in their occupations. Among individuals who were employed prior to falling ill, the debilitating effect of advanced HIV/TB infections forced participants to cease working. Most of these participants noted that they experienced changes in their energy levels:

“I do not quite have the same strength to work as I used to” (Male, 31, P6); “I have stopped now because I am sick, I have no energy that’s needed to work” (Female, 45, P9).

Loss of the physical ability to conduct work was a threat to HRQOL as it impacted on participants’ livelihood. Loss of employment meant loss of income and an increased dependency on others. All participants spoke about being dependent on others for financial or material assistance after falling ill.

#### Self-care

Living with HIV and TB made patients more cognisant of their health and awakened a sense of responsibility to attempt to maintain good health, which sometimes required them to make lifestyle changes. Participants indicated that to maintain good HRQOL involves paying attention to one’s needs, prioritising health, introduction of exercise and a healthy diet and to abstain from alcohol use to keep the body healthy. Phrases such as,

“It is to look after yourself and taking good care of yourself” (Male, 31, P1); “I must look after my health” (Female, 28, P2); “…requires taking good care of health like exercising, keep a proper diet and look after your wellbeing” (Female, 32, P5); were common.

At the time of the study, study participants were on treatment for TB, which would have involved daily pill-taking. As recent initiation on to ART was a primary eligibility criterion for inclusion in the study, none would have been on ART for an extended period. As the study was an RCT, neither staff nor participants would have known about the inclusion of prednisone in their treatment regimens. Given the volume of medication participants were taking, it was unsurprising that not all participants could recall what medication they were taking, or the duration required to keep taking it. They were, however, aware that taking medication was important to their health and HRQOL. Irrespective of the medications they were taking, participants were advised that they should abstain from drinking alcohol. Many spoke about finding this difficult as they equated one form of self-care as being able to do what they did before their diagnoses, including drinking alcohol with friends. Many participants perceived self-care in the form of abstinence from alcohol as a threat to HRQOL because they saw consuming alcohol with friends as an activity which promoted a good quality of life. Not being able to participate in social events/alcohol consumption acted as a reminder that their quality of life was diminished because of their condition.

### Social experiences of HRQOL

Participants discussed the social domain of HRQOL as closely related to the physical experiences of HRQOL. They identified social experiences of HRQOL in two main ways. The first related to partaking in activities they had participated in prior to their illness (like drinking alcohol as a social activity mentioned above) that we have named “usual activities”, and the second whether and how they experienced stigma.

#### Usual activities

Usual activities revolved around two aspects: employment and socialising with peers. Employment was a notable dimension of HRQOL in the social domain; as one participant noted in response to the question how they understood good HRQOL:

“I would say it’s a person that doesn’t have any problems, that lives their own life, who has a job, like you for example”. (Male, 35, P15)

Being employed gave participants a source of income that they could use to financially support those in their immediate social networks. For many participants, living with HIV and TB, particularly in the acute phase of TB-IRIS, meant participants were too ill to perform the tasks associated with their employment, which often involved some level of physical strength (eg. construction work). As most participants were precariously employed, their positions and income would not have been protected if they were unable to perform their work tasks. Without income, participants were unable to fulfil their social role as breadwinners and felt despondent; as a male participant expressed “life is stuck now” when reflecting on the loss employment and income (Male, 35, P15).

In the absence of steady income, participants increasingly depended on others for financial support. This took away independence and autonomy in daily decision-making regarding, for example, acquiring food or planning meals. Participants further experienced this lack of autonomy as a personal failure to provide for their families and maintain a household. The pressure of social responsibility was also reflected in the perceived need to obtain employment. The following quote illustrates this:

“I was the first one to get a job, I left school, and I didn’t even go to high school. I was in standard 7 but dropped out halfway through and came to work here; because I saw that my brother was struggling”. (Male, 35, P16)

In addition, participants felt that autonomy was also taken from them in domains where HIV and TB treatment regimen impacted on engagement in social activities. In this regard, usual activities of socialising with friends over the consumption of alcohol was a major factor impacting on participants’ daily lives. Although the recommendation to patients was never that they should abstain from using alcohol, it is recommended that if they were to use alcohol, they should only do so moderately as intoxication could lead to failing to adhere to treatment. Many participants took this to mean that they are not permitted to use alcohol. Following, participants searched for loopholes in consuming alcohol along with treatment.

It became evident that much of socialising behaviour revolved around the use of alcohol. One participant discussed his life prior to falling ill as:

“It was nice, I enjoyed my life, we would drink and get drunk every weekend, I would enjoy myself, would always be around girls but things changed when I realised, I was ill”. (Male, 31, P1)

Participants discussed scheduling of medication and attempted to identify time periods where the consumption of alcohol would not induce severe side effects or negatively impact on health outcomes.

Throughout interviews participants frequently probed the interviewer on the use of alcohol along with being on treatment. Participants enquired about the potential effects of attempting to adhere to treatment and consume alcohol through sketching potential scenarios:

“…and let’s say I am unable to drink them (medication), because I have already had a sip of alcohol and, so I make a decision not to (drink them)?”; “So, it does not matter whether you mix them with alcohol?”; “So, it’s not a problem if I drink them from Monday to Friday, then Saturday I drink alcohol and drink them as well?” (Male, 31, P1; Female 28, P2).

“There is a friend of mine…okay my friend’s friend. He eats ARVs and drinks, how does he do all of this on the days he drinks?”; “…let me say he normally drink(s) his pill(s) at 9 pm at night and he decides to drink alcohol during the day, can he still drink them (medication) at night?”. (Male, 34, P11).

However, some participants reported changes in lifestyle with HIV/TB and increased cognisance of health:

“It (my life) has now changed because I used to be someone who used to go to parties, drink alcohol and on weekends I would enjoy myself by drinking alcohol but now I know that I cannot drink, I have to look after my health”. (Female, 28, P2)

Another participant reflected a similar experience:

“For example, if I was at a party, drinking with friends; I could drink for as long as I wanted or when the alcohol finishes but now even if I am out, I must always be mindful of the time I am supposed to take my treatment so that I do not miss it”. (Male, 33, P3)

#### Stigma

Stigma was a theme present within the social domain in the context of employment, disclosure to family, and perceived stigma in the larger community. Employed participants were required to discuss their condition with their employers as they may need to miss work to attend clinic appointments and would often be too ill to undertake work-related tasks. We did not ask whether this always included HIV disclosure, but for some participants it did. Telling employers about their illness led to fear of the potential risk of experiencing HIV/TB associated stigma from employers. Their disclosure was well received by employers and there were attempts to accommodate them and their health states.

“I had to tell my boss about the situation (illness and treatment) and he was understanding”. (Male, 31, P1)

Participants’ disclosure to family members, such as parents/caregivers and spouses/partners were also well received. As one participant stated after disclosing his HIV status:

“Nothing has changed at home. Things are still good”. (Male, 33, P3)

All participants experienced support from those close to them. Support came in the form of moral support, assistance with travel and material needs as well as financial aid.

“…my child takes care of me and gives me things like money”; “Yes, she and her husband [support me]. So, I did not see any difficulties, because each month they give me money. They try to ensure that I am not stressed. They do everything for me when I have a need”. (Female, 48, P7)

While participants received acceptance and support from close associates, the role of stigma was present in the participants’ narratives in various ways. Some of the statements that participants made indicated that they did not experience stigma, as effective treatment allowed those living with HIV/TB to present as others do:

“I mean to say you cannot separate from those who do not have these things (HIV and TB). I am okay, I also look like them (people not living with HIV/TB). The only difference is that I eat treatment, and no one can tell that I do”. (Female, 45)

However, secrecy regarding taking treatment for HIV and TB points to some level of fear of discrimination and anticipated stigma from others. Perceived stigma was further experienced in the larger community. Stemming from a fear of being stigmatised one participant moved closer to and preferred to receive treatment at a different clinic further away as opposed to the HIV treatment facility close to her home.

“That side they still view HIV people as.… so, I feel like they will make me not feel okay whereas this side I feel like people are more open”;

“They look down on someone with HIV, make them feel ashamed”. (Female, 45, P9)

Although not articulated explicitly, it was clear from participants’ responses that they had also internalised some of the stigma they felt was directed at them from others. This also affected their mental state, which we discuss in more detail below.

### Mental experiences of HRQOL

#### Mental health concerns

Participants’ mental health concerns manifested as worry, fear and stress. Participants’ anxiety and concerns were largely expressed in relation to their social networks. Participants worried about the wellbeing and futures of children, siblings, and caregivers in the event of their death. Participants expressed apprehension about the care and financial support of their loved ones should they not recover from their illness. As one participant expressed her fear confronted with uncertainty towards the future:

“My concern was leaving my child behind at a young age so those are the kind of things I would think about. But it was explained to me that if I take my treatment regularly then I would be able to see my child grow. That is what was concerning me”. (Male, 33, P3)

Another participant was fearful of how her illness might impact her children emotionally.

“It’s fear. That if I were to tell them they might feel sad that their mother might die early” (Female, 45, P9).

Similar concerns were noted by another participant who posed a question about care to the interviewer:

“The one thing I would like to know is that, should I get sick again, would you be able to assist with my siblings who are in school?”. (Male, 31, P6)

Another female participant struggled to cope with the worry she witnessed in her children once they knew about her condition to the extent that she sent her children to live with her mother:

“I was very worried about them and I would also just cry because I felt hurt and most times I would send them to stay with my mom because they would always cry when they saw how I was; so I wanted to remove them from that environment”. (Female, 32, P5)

For some participants, stress was directly related to their ability to earn an income and care for themselves. As a 48year-old female explained:

“…even though I experienced some stress, but I told myself that I do not want to hinder myself from healing by focusing too much on the fact that I am sick and I do not work…” (Female, 48, P7).

For others stress was related rather to having to adhere to daily medication than to being ill itself:

“It’s stressful to have both (HIV and TB), particularly having to take pills every day…. and sometimes you forget to do so.” (Male, 31, P1)

#### Coping

Within the mental domain, participants mentioned coping with their diagnoses as an important aspect that affects HRQOL. Some participants found strength in the notion that they were not the only ones infected by HIV and TB:

“I learned to accept and told myself that these diseases do not belong to animals, they belong to people, and I am not the only person who has them. It will be different and problematic if they were rare diseases, a case whereby nothing is known about them and they are still being researched. Mine are well known so I have no problem, I just accepted things as they are for me to live with them and take treatment”. (Male, 31, P8)

Others coped with their illness with the knowledge that medication can help them live lives of similar quality to those not living with HIV/TB, which gave them hope:

“…living with both HIV and TB is not the end of the world. There is a lot that one can still do and take medication regularly it is manageable, and I can live the same life as anyone else” (Female, 32, P5).

## Discussion

We set out to explore the subjective meaning of HRQOL among people living with HIV/TB co-infection, and the perceived impact on several life domains considered important for HRQOL in an impoverished, peri-urban informal settlement of Khayelitsha, South Africa. Participants spoke about HRQOL in relation to their physical well-being, how being ill caused them to lose physical strength and how they attempted to care for themselves to address this. In relation to the social dimensions of HRQOL, participants spoke about their (in)ability to engage in ‘usual’ activities, and the role of stigma in their experience of HRQOL. In relation to mental health, they spoke about their struggles to cope with their conditions, mainly influenced by social factors.

Although HRQOL is a measure that seeks to unpack individual experiences, we found that the social experience of HRQOL emerged as the common denominator connecting the individual domains of experience. Using IPA produced findings that echo how health sociologists have viewed health as a political and social issue, where the organization of societal resources greatly impacts how individuals experience health [40]. Socially stigmatized diseases such as HIV and TB, as evidenced by our data, clearly show the relationship between social and structural determinants of health and individual agency, which has also shaped also the HRQoL as perceived by our participants.

Managing conditions like HIV and TB have far reaching implications in social settings and within the social relationships of those afflicted. The ways that these conditions are negotiated through social relationships can affect the spread of and distribution of the infection, as well as the social response in terms of support, stigma and subsequently help seeking behaviour and treatment adherence [32, 33]. Patients are not passive passengers on a treatment journeys, but instead enact their lived experience of their illness and assign meaning to it [32]. Using IPA furthered our understanding of what is at stake for patients as it moves beyond what can be more positivist and singular biomedical perspectives.

The social experience and consequences of this loss of physical strength was of greatest concern to participants, typified by their inability to continue to work. Participants tended to hide their illness from employers for as long as they could to prolong their receipt of income and limit financial reliance on others. Unemployment and needing additional social support created uncertainty and confusion for participants. Some questioned their role in society and worried about who would care for their loved ones, particularly their children, if they remained unable to provide including in the event of their death [41, 42]. This echoes research that underscores that employment offers an integral source of structure to life, a network of social support, a sense of belonging, a role to fulfil and adds meaning to life [43]. Employment is associated with improved HRQOL [6, 44] as it encourages interaction with peers and establishes social life growth while strengthening social support [45].

In addition to wanting to prolong employment, participants delayed disclosing their health status to employers as they feared they would be stigmatized. The effect of stigma on people living with HIV has been well-documented as having a negative impact on access and adherence to treatment [46, 47], physical and mental health [47–49] and reduce HRQOL [47, 48]. Patients’ physical experiences of health have profound effects on HRQOL due to the interwoven social experience of the potential stigma associated with illness disclosure.

The increased awareness of health status and self-care to preserve health was also experienced socially. Participants expressed a desire to adhere to social norms and expectations through trying to adopt self-care routines that might allow them to hide the fact that they were ill. But daily pill-taking served as a painful reminder of the fact that they were not in the same health as others. But for those who had been adherent to treatment for some time, they were relieved that their pill taking meant that their illnesses would not be physically visible to others [50]. As Kagee, Swartz and Swartz [51] theorise, ART adherence cannot be understood as an individual endeavour, as engaging with HIV treatment is shaped by multiple social, historical, political and structural factors that can act as barriers or facilitators to sustained engagement with ART.

Living with HIV/TB, as well as negotiating unemployment, pill-taking, and experiences of stigma all created stress for participants. Several also spoke about the difficulty of having to limit alcohol consumption, which had previously played a central role in their social lives [52, 53]. In a high alcohol consumption setting such as Khayelitsha, the use of alcohol as a coping mechanism is not restricted to illness but could also be connected to living in an impoverished context where people face significant socio-economic challenges [54]. Alcohol consumption may act as a coping strategy in two ways: for its self-medicating suppressant effects [55], and as a link to the social experience of consuming alcohol with peers that in the past produced a sense of well-being and belonging [52]. Although none of the participants in our study reported alcohol consumption during the study period, many attempted to establish whether using alcohol and medication was at all possible Some tried to find ways to take medication and drink alcohol at different times of the day in attempt to reclaim part of their social autonomy [56, 57]. Despite ambiguity in literature regarding the protective effects of social support and alcohol consumption [55] most participants did express good social support suggesting that, in this setting, social support might act as a protective measure to alcohol consumption among those living with HIV and TB.

In relation to the social and collective experience of health and illness, it was potentially helpful for participants to know that they are not alone in living with HIV/TB, especially in a high prevalence area like Khayelitsha. This sense of universality could allow people to regard HRQOL not solely as an individual or isolating experience of being ill, but rather as a way of sharing a commonality with others, even those unknown to them. Universality could be experienced as a sense of belonging among those living with HIV, which in turn has been shown to mitigate the effects of stigma [52, 58, 59].

A sense of belonging through social interactions has further been shown to be a positive predictor for good HRQOL, impacting in particular the physical and mental domains [60–62]. Furthermore, social connections, interactions and social support improves resilience among patients living with TB and mental health among patients living with HIV [63].

Recently, HRQOL has also gained attention in international HIV policy and monitoring frameworks. The “fourth 90” was proposed, but not yet adopted [19] due to difficulties in finding a consensus on how to operationalise and measure this construct for global policy targets. In this sense, our findings provide an important contextualised in-depth understanding of what HRQOL means to people living with HIV and TB in a South-African context, and they may inform the development of qualitative indicators of HRQOL among people living with HIV/TB co-infection.

## Strengths and weaknesses

The findings of this paper should be interpreted with the following limitations in mind. Participants were recruited purposefully from the trial sites in Khayelitsha. Eligibility criteria to patient’s enrolment into the PredART trial stipulated that they had to have a CD4 cell count of less than 100 cells per microliter indicating severe immune suppression which meant that they were often potentially physically and socially more disadvantaged than those with higher CD4 cell counts. As potential participants were often very ill, we were unable to recruit as many participants as we had originally hoped, thus making our findings less diverse.

As indicated, this qualitative study was nested in a treatment trial, which prevented us from fully achieving the following widely used quality criteria to establish trustworthiness [64]. Although some interviews were rather short, data collectors engaged fully with the study participants, resulting in rich data. Trained interviewers established good rapport and trustful relationships with the participants, thus ensuring a rigorous data collection process enhancing the credibility of our findings. Using a detailed study protocol, standard operating procedures, making interview topic guides and log-books available to track the data collection process, contributed to the study’s dependability. The coding process was done through a team effort, and the team reached consensus on codes and final themes. When analysing the data, the team used self-reflective techniques through regular meetings to debrief after data collection and made necessary changes if needed. The team adopted investigator triangulation to increase confirmability (i.e. decision making through collaboration, discussion and participation of the qualitative study team reaching consensus on different perspectives). Finally, we acknowledge that the degree to which the results can be generalized or transferred to other contexts or settings is limited. Although we used a purposive sampling technique, we could not achieve data saturation due to the limited sample within the larger trial study. In this regard we refer to recent debates in qualitative research acknowledging that meaning is always subjectively interpreted and not excavated from the data [65] leading to flexible data saturation approach. Due to a potential social desirability bias, participants might have responded in a manner they believed to be appropriate in terms of treatment and self-care which could have had an unknown bias on HRQOL responses.

## Conclusion

This study’s findings advance our in-depth understanding of HRQOL among people living with HIV-associated TB in resource-constrained settings such as Khayelitsha. The findings illustrate the centrality of the social experience of HRQOL in relation HIV-associated TB. This study contributes to a more nuanced understanding of how people in a resource constrained setting conceptualise HRQOL and could therefore inform future interventions to improve their HRQOL. We draw particular attention to the need to consideration for people with HIV and TB as comorbidities- a key and growing challenge for health systems and public health programming to address in future. For future research we recommend greater integration of social aspects into the understanding of HRQOL as patient-reported outcome measures in medical and public health decision-making, as well as the development of patient empowering guidelines and resources to address social aspects of health.

## Supporting information

S1 Appendix Debriefing interview topic guide. (DOCX)
