# Supplementary material for: Exploring Health-Related Quality of Life (HRQOL) among patients with HIV-associated TB in Khayelitsha, South Africa
Source: PLoS One. 2024 Nov 4;19(11):e0275554. doi: 10.1371/journal.pone.0275554 (PMC11534229; doi:10.1371/journal.pone.0275554)
Supplement: S1 Appendix — (DOCX) [file pone.0275554.s001.docx]

**PredART QoL sub-study Debriefing Interview Topic Guide**

**Section one: What does “quality of life” mean to participants in the study?**

1. What do you think a good “quality of life” is?
2. Could you describe time when you thought your quality of life was good?
3. What sorts of things do you think positively affect the quality of your life?
4. What sorts of things negatively affect your quality of life?
5. How has having HIV affected your quality of life?
6. Which factors linked to being HIV positive have affected your quality of life the most?
7. How has having TB affected your quality of life?
8. Which factors linked to having TB have affected your quality of life the most?
9. Which symptoms have affected your quality of life the most?
10. How have you managed the factors affecting your quality of life?
11. Who has been most involved in this process with you?

**Section Two: Do you think that our QoL instruments captured your perceptions and understandings of “quality of life”?**

1. How did you feel about filling out the 3 QoL instruments?
2. How do you feel about the questions asked in the forms?
3. Were there any questions that you think were not asked that should have been?
4. Which other questions could we have included?
5. Were there any questions that you think were asked that should not have been asked?
6. Which questions might you have left out of the questionnaires?
7. From your responses to the 3 questionnaires, do you think we can better understand how you see “quality of life”? Why do you say this?

**Section Three: Which instrument do you think best captured your experience of quality of life?**

1. Which questionnaire asked the most relevant questions to your understanding of quality of life?
2. Which questionnaire asked the least relevant questions to your quality of life?
3. If you were to design one form, which combination of questions would you include in it?
